# The zebrafish as a tool to identify novel therapies for human cardiovascular disease

**DOI:** 10.1242/dmm.016170

**Published:** 2014-07

**Authors:** Aarti Asnani, Randall T. Peterson

**Affiliations:** Massachusetts General Hospital Cardiovascular Research Center, Harvard Medical School, Charlestown, MA 02129, USA.

**Keywords:** Cardiovascular, Drug discovery, Zebrafish

## Abstract

Over the past decade, the zebrafish has become an increasingly popular animal model for the study of human cardiovascular disease. Because zebrafish embryos are transparent and their genetic manipulation is straightforward, the zebrafish has been used to recapitulate a number of cardiovascular disease processes ranging from congenital heart defects to arrhythmia to cardiomyopathy. The use of fluorescent reporters has been essential to identify two discrete phases of cardiomyocyte differentiation necessary for normal cardiac development in the zebrafish. These phases are analogous to the differentiation of the two progenitor heart cell populations in mammals, termed the first and second heart fields. The small size of zebrafish embryos has enabled high-throughput chemical screening to identify small-molecule suppressors of fundamental pathways in vasculogenesis, such as the BMP axis, as well as of common vascular defects, such as aortic coarctation. The optical clarity of zebrafish has facilitated studies of valvulogenesis as well as detailed electrophysiological mapping to characterize the early cardiac conduction system. One unique aspect of zebrafish larvae is their ability to oxygenate through diffusion alone, permitting the study of mutations that cause severe cardiomyopathy phenotypes such as *silent heart* and *pickwick*^m171^, which mimic titin mutations observed in human dilated cardiomyopathy. Above all, the regenerative capacity of zebrafish presents a particularly exciting opportunity to discover new therapies for cardiac injury, including scar formation following myocardial infarction. This Review will summarize the current state of the field and describe future directions to advance our understanding of human cardiovascular disease.

## Introduction

The zebrafish has emerged as an important vertebrate model for the study of cardiovascular disease. Zebrafish larvae are transparent, allowing easy visualization of the heart and vasculature for high-throughput phenotypic assays. Fluorescent markers can be used *in vivo* to tag specific cell types and visualize their location and migration during embryogenesis. Moreover, sophisticated videomicroscopic techniques have been developed for quantification of ventricular function and for heart rhythm analysis. Zebrafish are particularly well-suited to genetic manipulation to simulate human cardiac disease, and of the disease-related genes listed in the Online Mendelian Inheritance in Man (OMIM) database, 82% have at least one zebrafish ortholog (Howe et al., 2013). In contrast to traditional methods of mutagenesis, injection of morpholino oligonucleotides permits much more rapid targeted gene knockdown. New genome-editing tools such as TALENs (transcription activator-like effector nucleases) and CRISPR (clustered regularly interspaced short palindromic repeats)/Cas have also been applied to the zebrafish model, providing exciting new opportunities for high-efficiency mutagenesis (Bedell et al., 2012; Hwang et al., 2013; Sander et al., 2011).

Given the ease and rapidity with which the zebrafish genome can be manipulated, the zebrafish is uniquely positioned among vertebrate models as a platform for chemical genetics. Small-molecule screening has been used to identify novel suppressors of disease phenotypes and characterize associated molecular pathways. In addition, zebrafish can be used to ‘functionalize the genome’; in other words, to provide insight into the biological function of the many candidate genes being rapidly identified in human genome-wide association studies (Kettleborough et al., 2013). Here, we review examples of these emerging techniques as applied to zebrafish models of human cardiovascular development and disease, and highlight opportunities for these models to facilitate further discovery in these areas.

## Drawing parallels between the zebrafish and human cardiovascular systems

### Heart development

In the two-chambered zebrafish heart, deoxygenated blood enters the sinus venosus and passes through a single atrium and ventricle. The ventricle then pumps venous blood through the bulbus arteriosus to the ventral aorta for distribution to the gills for oxygenation. Despite clear anatomic differences between the zebrafish heart and four-chambered mammalian heart, several studies have highlighted similarities in the genes and regulatory networks driving cell fate. In mammals, early cardiomyocytes contributing to the formation of the left ventricle arise from a collection of progenitor cells located in the first heart field, whereas the subsequent addition of cardiomyocytes to the outflow tract occurs via a second and distinct cell lineage located in the second heart field (Buckingham et al., 2005). In zebrafish, real-time studies using fluorescent markers have furthered the concept of the first and second heart fields as distinct progenitor cell populations similar to those seen in mammals. To create a timeline for cardiomyocyte differentiation in the developing heart, a double-transgenic zebrafish model was developed to express the fluorescent markers eGFP and dsRed driven by the cardiac-specific *cardiac myosin light chain 2* (*cmlc2*) promoter (de Pater et al., 2009). In contrast to rapidly fluorescent eGFP, dsRed requires at least 24 hours to mature, and a change in fluorescence pattern can be used to distinguish early and late developmental effects. In this model, two distinct phases of cardiomyocyte differentiation were identified. First, the ventricle and part of the atrium (venous pole) were created. Next, cardiomyocytes were added to the arterial pole, which later developed into the outflow tract. This chronology was subsequently confirmed using a cardiac-specific red-to-green photoconvertible fluorescent protein. Injection of antisense morpholinos against *islet1* (*isl1*) and *fgf8*, genes implicated in mammalian heart development, demonstrated that Isl1 is required for cardiomyocyte differentiation at the venous pole, whereas FGF8 signaling is necessary for cardiomyocyte addition at the arterial pole. Furthermore, this second phase of differentiation is dependent on the expression of *mef2cb*, analogous to the second heart field marker *mef2c* seen in the mouse (Lazic and Scott, 2011).

**Fig. 1.**
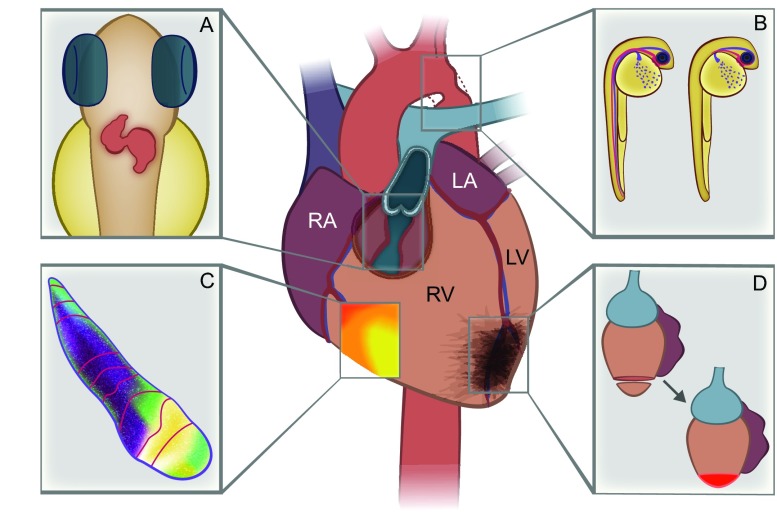
**The zebrafish as a model to study human cardiovascular disease.** Middle panel shows the four-chambered human heart with associated cardiovascular pathologies. RA, right atrium; LA, left atrium; RV, right ventricle; LV, left ventricle. Side panels show examples of cardiac development and disease studied in the zebrafish. (A) In contrast to the human heart, the zebrafish heart is two-chambered with a single atrium and ventricle. Characterization of the second heart field in zebrafish has the potential to illuminate new therapies for human congenital heart defects such as right ventricular outflow tract obstruction. (B) Zebrafish expressing the *gridlock* mutation fail to develop circulation in the tail and trunk (wild-type fish on left; *gridlock* fish on right), similar to human aortic coarctation. (C) Calcium activation in the zebrafish heart mimics wave propagation in the human heart, enabling the study of arrhythmias. Isochronal mapping of the zebrafish ventricle using a fluorescent calcium indicator (color gradient) demonstrates calcium excitation over time, with red lines depicting areas of the ventricle depolarized simultaneously. (D) Zebrafish hearts regenerate following injury, a process that could be harnessed therapeutically to prevent scar formation following myocardial infarction. Red cells designate regrowth of cardiomyocytes in the zebrafish.

The characterization of two discrete phases of cardiomyocyte differentiation in the zebrafish heart has important implications because many of the genes and regulatory pathways essential to embryogenesis are conserved between zebrafish and mammals. Compared to other vertebrate models, zebrafish larvae are more amenable to *in vivo* imaging experiments because of their transparency and external development, allowing a more comprehensive description of cardiomyocyte fate. Such studies offer insight into developmental pathology and have the potential to define new treatment strategies for human congenital heart disease (Fig. 1A). For instance, many individuals with DiGeorge syndrome exhibit conotruncal heart defects that have been attributed to heterozygous deletion of the chromosomal segment 22q11.2. This segment contains the *TBX1* gene, which encodes a key transcription factor in embryonic development. In zebrafish, Tbx1 is necessary for proliferation of the second heart field, and *tbx1*-null embryos mimic the heart defects seen in DiGeorge syndrome (Hami et al., 2011; Nevis et al., 2013; Piotrowski et al., 2003). Further characterization of the *tbx1*-null zebrafish model has the potential to provide insight into early markers of conotruncal defects and identify opportunities for therapeutic intervention.

Similarly, the homeodomain transcription factor Nkx2.5 is necessary for initial development of the ventricular myocardium in murine models, although its role in maintenance of chamber identity was not previously defined (Biben et al., 2000; Lyons et al., 1995; Yamagishi et al., 2001). In zebrafish, mutation of *nkx2.5* results in overgrowth of the atrial chamber and a reduction in size of the ventricular chamber (Targoff et al., 2013). Moreover, loss of both zebrafish homologs, *nkx2.5* and *nkx2.*7, leads to gradual elimination of all ventricular cardiomyocytes (Targoff et al., 2013). During this process, expression of the ventricular gene *vmhc* decreases, whereas expression of the atrial gene *amhc* increases, suggesting that Nkx2.5 is necessary for maintenance of ventricular identity. In humans, mutations in NKX genes have been identified in individuals with common congenital heart defects, including atrial septal defect, outflow tract abnormalities, hypoplastic left heart syndrome and atrioventricular (AV) block (Benson et al., 1999; Elliott et al., 2003; McElhinney et al., 2003; Schott et al., 1998). Efforts to identify small molecules affecting these transcriptional pathways in zebrafish could yield novel therapies for congenital heart disease as well as for adult cardiac pathology.

### Vascular development

Vasculogenesis and angiogenesis have long been recognized as being complex and tightly regulated processes. Many of the biochemical pathways involved in embryonic vascular development have been implicated in adult human disease, including pulmonary arterial hypertension, hereditary hemorrhagic telangiectasia and preeclampsia (Lane et al., 2000; Johnson et al., 1996; Venkatesha et al., 2006). As with studies of heart development, the use of fluorescent reporters in zebrafish allows for direct visualization of vascular development *in vivo*, offering a complementary approach to *in vitro* endothelial cell sprouting experiments. For instance, a transgenic *fli1:eGFP* zebrafish model, in which the entire vasculature is green, has been used to investigate the role of extracellular bone morphogenetic protein (BMP) modulators in angiogenesis (Heinke et al., 2013). In this model, the proteins BMP endothelial cell precursor-derived regulator (BMPER) and twisted gastrulation (Tsg) were essential for development of an intact vascular system. Injection of morpholinos targeted against BMPER and Tsg resulted in obvious venous malformations involving the tail vein, intersomitic vessels and caudal vein plexus. The exact role of these extracellular modulators remains to be defined, and complementary techniques using selective inhibitors of BMP and its downstream targets [Smad, mitogen-activated protein kinase (MAPK)] have the potential to provide a more complete characterization of early angiogenesis (Sanvitale et al., 2013; Yu et al., 2008). Likewise, inducible transgenic zebrafish models using heat-shock promoters, for instance, could be valuable in dissecting the temporal role of BMP signaling in vascular development (Wiley et al., 2011).

Direct visualization of vascular phenotypes in zebrafish has also enabled high-throughput small-molecule screening to elucidate the mechanisms behind congenital vascular defects. Zebrafish expressing the gridlock mutation *grl* in the *hey2* gene exhibit malformation of the aorta (Weinstein et al., 1995). As a result, there is disruption of circulation to the tail and trunk and development of collateral vessels, analogous to human aortic coarctation (Fig. 1B). Using this model, a chemical screen of 5000 candidate small molecules identified two structurally similar compounds that rescued the gridlock phenotype in a dose-dependent manner, resulting in normal flow through the aorta (Peterson et al., 2004). Subsequent experiments demonstrated that these drug candidates upregulate expression of vascular endothelial growth factor (VEGF), which plays a crucial role in formation of the aorta. These types of chemical suppressor screens offer insight into the molecular pathways responsible for developmental vascular defects such as aortic coarctation.

In addition, chemical screening in zebrafish can be used to inform our understanding of complex vascular processes such as atherosclerotic plaque formation. In a large screen of compounds affecting dorsoventral axis formation, dorsomorphin was identified as a small-molecule inhibitor of BMP signaling (Yu et al., 2008). An optimized analog of dorsomorphin, LDN-193189, inhibited the development of atheroma in low-density lipoprotein (LDL)-receptor-deficient mice (Cuny et al., 2008; Derwall et al., 2012). Treatment with LDN-193189 also resulted in decreased vascular calcification and inflammation as well as decreased LDL cholesterol levels. Zebrafish models have successfully replicated other vascular phenotypes such as AV malformations and tumor neovascularization (Corti et al., 2011; Nicoli and Presta, 2007), providing opportunities for similar high-throughput experiments to identify new drug targets.

### Valvular development

Studies describing valvulogenesis in zebrafish have contributed to the identification of epigenetic factors that might be dysregulated in human valvular disease. In a comprehensive histological analysis in zebrafish, development of the AV valve leaflets from the endocardial cushion occurred during the larval period, between 6 and 16 days post-fertilization (dpf), and was characterized primarily by cell migration rather than cell proliferation (Martin and Bartman, 2009). Subsequent valve maturation was influenced by animal size, suggesting that hemodynamic forces have the potential to modulate valve thickness and length. The timeline and early mechanisms of valve development differ in zebrafish when compared with the developing mouse or chick (Combs and Yutzey, 2009). Nonetheless, the molecular regulators and hemodynamic contributions to valve formation seem to be conserved between these vertebrate models. For example, zebrafish valve formation is dependent on Notch and Nuclear factor of activated T-cells (NFAT) signaling, as seen in prior murine models (Beis et al., 2005). Zebrafish with the *silent heart* mutation lack heart contraction and fail to develop a normal valve shape, highlighting the role of cardiac contractility and shear stress in this process (Bartman et al., 2004). These types of models could be useful for identifying novel modifiers of valvulogenesis to serve as druggable targets for human congenital valve disease.

Recently, zebrafish models have focused on microRNA (miRNA) regulation of valve formation. In a zebrafish *dicer* mutant lacking mature miRNAs, endocardial cushion formation was excessive, prompting the observation that miRNA-23 is necessary to restrict the number of cells that differentiate into endocardial cushion cells (Lagendijk et al., 2011). Similarly, miRNA-21 is required for normal valvulogenesis in the zebrafish, and its expression is highly dependent on blood flow (Banjo et al., 2013). The gene *pdcd4*, encoding programmed cell death protein 4, was found to be an important *in vivo* target of miRNA-21, and inhibition of miRNA-21 binding to *pdcd4* resulted in failure of atrioventricular valve development (Kolpa et al., 2013). Thus, zebrafish models have the potential to contribute to our understanding of cardiac valve development and pathology.

### Arrhythmia

The transparency of zebrafish embryos permits detailed optical mapping to characterize the cardiac conduction system (CCS). In a transgenic zebrafish line expressing a cardiac-specific fluorescent calcium indicator (cmlc2:gCaMP), four distinct CCS developmental stages were identified (Chi et al., 2008). Beginning at 24 hours post-fertilization (hpf), conduction traveled linearly and unidirectionally across the heart tube from the sinus venosus to the outflow tract, suggesting that the zebrafish has a sinoatrial (SA)-node-type pacemaker similar to that seen in humans. Between 36 and 48 hpf, a significant conduction delay develops in the AV canal, which separates the atrium and ventricle. As ventricular trabeculations form, a fast conduction network begins to develop within the ventricle. In this model, maturation of the fast conduction network resulted in apex-to-base conduction, although base-to-apex conduction has also been observed in zebrafish (Jensen et al., 2012). Based on their observations, Chi and colleagues (Chi et al., 2008) subsequently set out to identify regulators of CCS development using an ethylnitrosourea (ENU) mutagenesis screen. Seventeen conduction-specific mutations were identified, including the homeobox transcription factor gene *tcf2*. In addition to the previously identified regulators of AV conduction (*notch* and *neuregulin*) (Milan et al., 2006), several new candidate genes were identified as potential therapeutic targets for cardiac conduction disease.

Similarly, zebrafish have been used to recapitulate type 2 long QT syndrome, which is caused by a genetic defect in the *KCNH2* gene that results in prolonged cardiac repolarization and life-threatening arrhythmias. As with the human disease, the zebrafish *KCNH2* mutant *breakdance* exhibits prolonged ventricular action-potential duration, spontaneous early afterdepolarizations and 2:1 AV block (two atrial contractions for each ventricular contraction), which is easily observed at the embryonic stage (Langheinrich et al., 2003). In a chemical screen of 1200 small molecules, the hit compounds flurandrenolide and 2-methoxy-N-(4-methylphenyl) benzamide (2-MMB) were identified as suppressors of the long QT phenotype (Peal et al., 2011). Through optical mapping experiments using a voltage-sensitive dye, these compounds were subsequently confirmed to shorten ventricular action potentials (Peal et al., 2011). Zebrafish models thus enable in-depth assessment and modulation of the electrophysiological properties of the heart, with the goal of identifying potential therapies for human arrhythmia (Fig. 1C).

### Cardiomyopathy

Several cardiomyopathy models have been developed in the zebrafish. Unlike other vertebrate models, zebrafish larvae have the unique capacity to oxygenate through diffusion alone for the first 7 dpf. This allows for the study of mutations leading to severe cardiovascular compromise, including those that result in a non-contractile heart. One such mutation, *silent heart*, was initially identified through a forward genetic screen and subsequently attributed to disruption of the gene *TNNT2*, encoding the cardiac contractile protein troponin T (Chen et al., 1996; Sehnert et al., 2002; Stainier et al., 1996). Zebrafish expressing the *silent heart* mutation also demonstrate a reduction in α-tropomyosin and cardiac troponins C and I, resulting in severe sarcomere defects and cardiomyocyte disarray. These ultrastructural changes parallel those seen in human *TNNT2* mutations, which lead to familial forms of hypertrophic and dilated cardiomyopathy. Similarly, the *pickwick*^m171^ mutation in zebrafish causes a weakly contractile heart and pericardial edema (Driever et al., 1996). This mutation was subsequently attributed to an alternatively spliced exon of the gene *ttn*, encoding the protein Titin, which is necessary for proper sarcomere assembly in cardiomyocytes (Xu et al., 2002). It has since become clear that *ttn* truncating mutations are an important cause of human idiopathic dilated cardiomyopathy, responsible for 25% of familial idiopathic cases and 18% of sporadic cases (Herman et al., 2012).

Similarly, a zebrafish model has been created to simulate amyloid light-chain amyloidosis, a plasma cell disorder that causes rapidly progressive cardiomyopathy (Mishra et al., 2013). Direct injection of human amyloid light-chain proteins into the zebrafish circulation results in a clear cardiomyopathy phenotype characterized by pericardial edema, cardiac dysfunction and cardiomyocyte death. Given the relatively low circulating blood volume in the zebrafish, only small amounts of light-chain protein are required to recapitulate systemic amyloidosis, an important advantage given the limited availability of human light-chain samples. In cultured cardiomyocytes exposed to amyloid light-chain protein, the development of cardiac dysfunction seems to be related to direct cardiotoxicity via non-canonical p38 MAPK activation (Shi et al., 2010). In zebrafish, this cardiotoxicity is replicated and can be rescued with administration of a selective p38 MAPK inhibitor (Mishra et al., 2013), suggesting opportunities for drug discovery for this rapidly progressive and often fatal disease.

Finally, treatment with cardiotoxic chemotherapies causes cardiomyopathy in zebrafish, analogous to human heart failure. Anthracyclines such as doxorubicin form a class of chemotherapeutic agents used to treat a number of common malignancies, including breast cancer, lung cancer, leukemia and lymphoma. They are extremely effective and form the backbone of many cancer treatment regimens used today. However, doxorubicin exposure causes dose-dependent cardiotoxicity, which can lead to clinically significant cardiomyopathy and congestive heart failure (Hershman et al., 2008; Lipshultz et al., 1991). Our group has found that administration of doxorubicin to zebrafish larvae recapitulates the heart failure phenotype, resulting in decreased myocardial contractility and increased cardiomyocyte apoptosis (Yan Liu and R.T.P., unpublished observations). Similarly, the widespread use of tyrosine kinase inhibitors for targeted treatment of cancer has led to concerns regarding their potential to cause cardiovascular disease, either through on-target or off-target mechanisms (Chen et al., 2008). Administration of the small-molecule tyrosine kinase inhibitors sunitinib and sorafenib results in ventricular dilation and impaired cardiac function in zebrafish larvae (Cheng et al., 2011). These models have the potential to aid in preclinical screening of chemotherapies, the identification of cardioprotective strategies during cancer treatment and, potentially, drug discovery for the broader heart-failure population.

### Regeneration

Given the limited treatment options for advanced heart failure, there is considerable interest in identifying ways to regenerate cardiomyocytes to restore myocardial function (Fig. 1D). In contrast to adult mammals, adult zebrafish can regenerate heart tissue following amputation of the ventricle (Poss et al., 2002). Using a Cre/*lox* system with a GFP reporter to track cardiomyocytes, this process was shown to occur predominantly through limited dedifferentiation of proliferating cardiomyocytes, rather than through stem or progenitor cells (Jopling et al., 2010). In this model, existing cardiomyocytes lost their sarcomeric structure, detached from one another and entered the cell cycle. These cells were found to abundantly express *gata4*, providing a molecular marker of regeneration (Kikuchi et al., 2010). In addition, Notch receptor expression is activated in the zebrafish endocardium and epicardium during regeneration (Zhao et al., 2014). Importantly, zebrafish models have been used to identify small-molecule modifiers of cardiac regeneration following injury. Using a transgenic zebrafish line with a fluorescent cell cycle indicator, a large chemical screen identified several small molecules along the Hedgehog, insulin-like growth factor and transforming growth factor-β signaling pathways that modulate cardiomyocyte proliferation during regeneration (Choi et al., 2013). These studies have contributed substantially to the field of heart regeneration, and zebrafish models will continue to remain important in illuminating new therapeutic strategies for end-stage cardiomyopathy.

## Conclusion

Because of its small size and ease of breeding, the zebrafish has become an invaluable model for the study of human cardiovascular disease. There remain countless opportunities for high-throughput chemical screening to both elucidate biology and identify small-molecule modulators of disease-associated pathways. In addition to furthering our knowledge of cardiovascular pathology, the unique ability of zebrafish cardiomyocytes to regenerate offers a particularly exciting opportunity to develop much-needed therapies for advanced heart failure. Given the ease of genome editing, particularly using new mutagenesis techniques such as CRISPR/Cas (Hwang et al., 2013), future studies will undoubtedly take advantage of the zebrafish model to pair human genetic mutations with their molecular functions. Improved screening techniques will facilitate this type of high-throughput investigation in zebrafish, adding to existing mammalian models as we advance our understanding of cardiovascular disease.
